# miR-375 and miR-30d in the Effect of Chromium-Containing Chinese Medicine Moderating Glucose Metabolism

**DOI:** 10.1155/2014/862473

**Published:** 2014-04-09

**Authors:** Qian Zhang, Xinhua Xiao, Ming Li, Wenhui Li, Miao Yu, Huabing Zhang, Fan Ping, Zhixin Wang, Jia Zheng, HongDing Xiang

**Affiliations:** Key Laboratory of Endocrinology, Ministry of Health, Department of Endocrinology, Peking Union Medical College Hospital, Peking Union Medical College, Chinese Academy of Medical Sciences, Beijing 100730, China

## Abstract

In China, TianMai Xiaoke tablet (TM) is used to treat type 2 diabetes. However, the exact mechanism of TM is not clear. This study is to investigate the effect of TM on glucose metabolism in diabetic rats and to identify whether TM takes a direct action through microRNAs on islet. Rats were divided into control group, diabetic group, low dose of TM group (TML), and high dose of TM group (TMH). Pancreas samples were analyzed using microRNA array and Q-PCR. Eight-week treatment with TM significantly decreased fasting blood glucose. The blood glucose was significantly reduced in TM-treated groups before and after oral glucose administration. Fasting insulin and HOMA-IR were suppressed in TM-treated groups. miR-448, let-7b, miR-540, miR-296, miR-880, miR-200a, miR-500, miR-10b, miR-336, miR-30d, miR-208, let-7e, miR-142-5p, miR-874, miR-375, miR-879, miR-501, and miR-188 were upregulated, while miR-301b, miR-134, and miR-652 were downregulated in TMH group. Through target gene analysis and real-time PCR verification, we found that these miRNAs, especially miR-375 and miR-30d, can stimulate insulin secretion in islet. Our data suggest that TM can improve blood glucose in diabetic rats which involved increasing the expression of miR-375 and miR-30d to activate insulin synthesis in islet.

## 1. Introduction

Diabetes mellitus (DM) is a chronic medical condition involving a group of metabolic disorders of multiple etiologies. It is characterized by hyperglycemia with disturbances in the metabolism of carbohydrate, fat, and lipid metabolism resulting from defects in insulin secretion, insulin action, or both. 90% of patients with DM belong to type 2 diabetes. DM is the fourth leading cause of death [1].

It is known that chromium deficiency will lead to impaired glucose tolerance due to insulin resistance and hyperglycemia [2]. Trivalent chromium is an essential mineral, which is thought to be necessary for normal glucose and lipid homeostasis [3, 4]. Trivalent chromium in a complex known as glucose tolerance factor, such as chromium picolinate, is considered the biologically active form.

TianMai (TM) Xiaoke Tablet comprises chromium picolinate (1.6 mg per tablet, equaling 200 *μ*g Cr),* Radix trichosanthis* (snake gourd root),* Radix ophiopogonis* (dwarf lilyturf tuber), and* Fructus schisandrae chinensis* (Chinese magnolia vine fruit) and in the ratio of 1.6 : 62.5 : 62.5 : 25. TianMai Xiaoke Tablet is approved by the State Food and Drug Administration of China (State Medical License no. Z20049007). TianMai Xiaoke Tablet can decrease HbA1c level [5].

MicroRNAs (miRNAs) are small noncoding RNAs of 18–25 nucleotides in length that bind to complementary 3′UTR regions of target mRNAs, inducing the degradation of transcriptional repression of the target [6]. miRNAs have been reported to regulate several metabolic pathways such as insulin secretion, cholesterol biosynthesis, and triglyceride, carbohydrate, and lipid metabolism [7–9]. Furthermore, not only have microRNAs been shown to be related to several human diseases, but also there is evidence that the modulation of miRNAs can provide therapeutic benefits [10–12].

However, little is known about the mechanism underlying the effect of TM through miRNAs. In the present study, we aimed to find the mechanism by which TM moderates hyperglycemia using miRNA arrays.

## 2. Materials and Methods

### 2.1. Animal Models, Grouping, and Treatment

Male Sprague-Dawley rats (280–320 g) were purchased from the Institute of Laboratory Animal Science, Chinese Academy of Medical Sciences and Peking Union Medical College (Beijing, China, SCXK-2012-0007). According to the previous study [13], diabetic models were fed a high-fat diet (40% of calories as fat, 41% carbohydrate, and 18% protein, Table 1) for 4 weeks and then administered with a single dose of streptozotocin (STZ, 50 mg/kg, tail vein) formulated in 0.1 mmol/L citrate buffer, pH 4.5 (Sigma-Aldrich). One week after STZ injection, the random blood glucose level of the diabetic rats was measured to confirm hyperglycemia. Random blood glucose above 16.7 mmol/L was used to define rats as diabetic. Diabetic rats were fed a high-fat diet throughout the experiment. Diabetic rats with a similar degree of hyperglycemia were randomly divided into three groups: vehicle, low dose TianMai Xiaoke Tablet (TML), and high dose TianMai Xiaoke Tablet (TMH) groups (*n* = 8, in each group). The typical human daily dose of TM is 480 mg/60 kg body weight. According to the following formula: *d*_rat_ = *d*_human_ × 0.71/0.11 [14], the corresponding dose of TM for rats is 51.64 mg/kg per day. Therefore, we selected 50 and 100 mg/kg per day as low and high dosages, respectively. The control (normal diet, 10% of calories as fat, 72% carbohydrate, and 18% protein, Table 1, *n* = 8) and vehicle group received 0.5% saline, whereas the TML and TMH groups were given TM (Hebei Fuge Pharmacy, China) at 50 and 100 mg/kg in 0.5% saline, respectively. The drug was administered once daily for 8 weeks using a gastric gavage. All animals were housed in an environmentally controlled room at 25°C in a 12 h light-dark cycle and were given free access to food and water throughout the experimental period. Fasting animals were allowed free access to water. After 6 weeks of treatment, an oral glucose tolerance test (OGTT) was performed. After 8 weeks of treatment, blood samples were taken from rats after anesthesia. Some pancreas tissue was then collected to perform the miRNA microarray and quantitative real-time PCR (qRT-PCR) experiments. All procedures involving animals were approved by the Animal Care and Use Committee of the Peking Union Medical College Hospital (Beijing, China, MC-07-6004) and were conducted in compliance with the Guide of the Care and Use of Laboratory Animals (NIH Publication no. 86-23, revised 1996). All surgeries were performed under sodium pentobarbital anesthesia, and all efforts were made to minimize suffering.

### 2.2. Measurement of Body Weight and Fasting Blood Glucose Levels

Body weight was monitored every 2 weeks. The 6 h fasting blood glucose (FBG) level was measured monthly using the enzyme end-point method (Roche, Germany) with blood from a tail bleed.

### 2.3. Oral Glucose Tolerance Test (OGTT)

After the rats had fasted for 6 hours, 2.2 g/kg of glucose was orally administered. Then, blood samples were collected from tail veins at 0 (prior to glucose load), 30, 60, and 120 min (after glucose load) for the glucose assay. The area under the curve (AUC) was calculated for blood glucose (BG) during the OGTT: AUC = 0.5 × (BG0 + BG30)/2 + (BG30 + BG60)/2 + 1 × (BG60 + BG120)/2.

### 2.4. Serum Biochemistry Analysis

At week 8, after euthanasia, blood samples were collected and centrifuged at 1000 g for 10 min. Serum was stored in aliquots at −80°C to assay serum fasting insulin, measured by enzyme-linked immunosorbent assay (ELISA, Millipore, USA); HOMA − IR = FBG  (mmol/L) × FINS  (*µ*U/mL)/22.5.

### 2.5. miRCURY LNA MicroRNA Array Experiment

miRCURY LNA miRNA Array contains 3100 capture probes, covering all rat microRNAs (388 miRNAs) annotated in miRBase 18.0, as well as all viral microRNAs related to rats. Total RNA was harvested from pancreas using TRIzol (Invitrogen) and miRNeasy mini kit (Qiagen) according to manufacturer's instructions. After having passed RNA quantity measurement using the NanoDrop 1000, the samples were labeled using the miRCURY Hy3/Hy5 Power labeling kit and hybridized on the miRCURY LNA Array v.18.0 (Exiqon). Following the washing steps, the slides were scanned using the Axon GenePix 4000B microarray scanner.

### 2.6. Gene Array Data Analysis

Normalization was performed by using a per-chip 50th percentile method that normalizes each chip on its median, allowing comparison among chips.

### 2.7. miRNA Quantitative Real-Time PCR (qRT-PCR)

Total RNA (5 ng) was reverse-transcribed using the TaqMan MicroRNA Reverse Transcription kit (Applied Biosystems) and the miRNA-specific reverse-transcription primers provided with the TaqMan MicroRNA Assay (Applied Biosystems). For the reverse transcription, a PTC-225 Peltier Thermal Cycler (MJ Research Inc., Waltham, Massachusetts) was used. The reaction was performed at 16°C for 30 min; 42°C for 30 min, and 85°C for 5 min. The obtained miRNA-specific cDNA was amplified using the TaqMan Universal PCR master mix II (Applied Biosystems) and the respective specific probe provided in the TaqMan Small RNA Assay (Applied Biosystems). PCR was performed using a CFX-96TOUCH (BIO-RAD). Amplification was performed at 95°C for 10 min, followed by 40 cycles of 95°C for 15 s and 60°C for 60 s. U6 small nuclear RNA was used as an endogenous control. The fold change in the miRNA level was calculated by the log⁡2 scale of the equation 2-ΔΔCt, where ΔCt = Ct miRNA-Ct U6 and ΔΔCt = ΔCt TMH samples − ΔCt DM samples [15].

## 3. Result

### 3.1. TianMai Xiaoke Tablet Showed No Effect on Body Weight of DM Rats

The mean body weight of DM rats decreased significantly compared with the control rats at week 2 (*P* < 0.05), week 4 (*P* < 0.01), week 6 (*P* < 0.01), and week 8 (*P* < 0.01). No significant differences were noted between the DM group and TM-treated groups (Table 2).

### 3.2. TianMai Xiaoke Tablet Decreased Fasting Blood Glucose of DM Rats

The fasting blood glucose (FBG) levels of DM rats were significantly higher than those of control rats at week 0 (*P* < 0.01), week 2 (*P* < 0.01), week 4 (*P* < 0.01), week 6 (*P* < 0.01), and week 8 (*P* < 0.01). FBG levels in TM-treated group decreased significantly at week 2 (*P* < 0.05), week 4 (*P* < 0.05), week 6 (*P* < 0.05), and week 8 (*P* < 0.05) compared to DM group (Table 3).

### 3.3. TianMai Xiaoke Tablet Moderated the Glucose Tolerance of DM Rats

**Figure 1:**
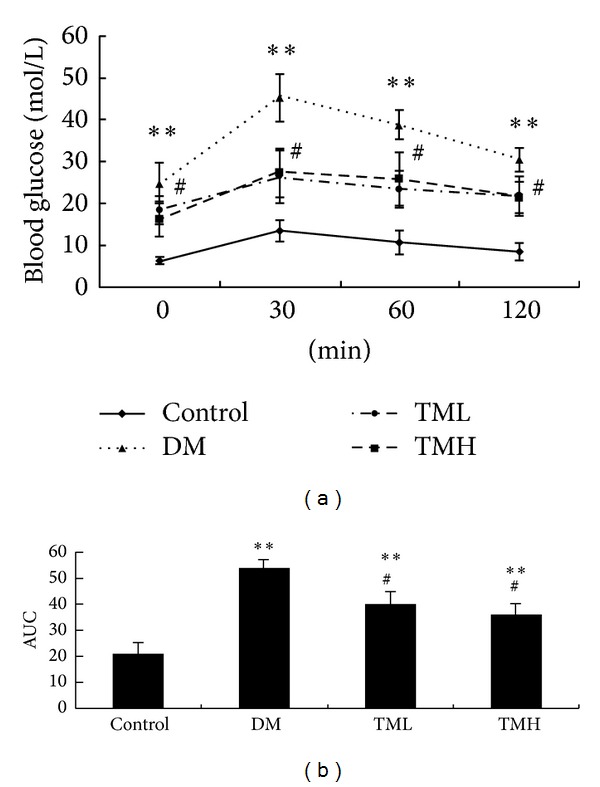
Oral glucose tolerance (mmol/L). TML: low dose of TianMai Xiaoke Tablet; TMH: high dose of TianMai Xiaoke Tablet. Data represent mean ± S.D. (*n* = 8). **P < 0.01 versus the control group. ^#^ *P* < 0.05 versus DM group.

The blood glucose levels in DM group were higher than those of control group before oral glucose administration (*P* < 0.01) and 30 minutes (*P* < 0.01), 60 minutes (*P* < 0.01), and 120 minutes (*P* < 0.01) after oral glucose administration. Blood glucose levels of TM-treated groups significantly decreased before and after oral glucose administration (*P* < 0.05, Figure 1).

### 3.4. TianMai Xiaoke Tablet Decreased FINS and HOMA in DM Rats

FINS and HOMA were significantly suppressed (*P* < 0.01) after 8-week TM treatment (Table 4).

### 3.5. miRNA Differentially Regulated by TM

In TMH group, 20 miRNAs showed a significant change (fold change > 2, *P* < 0.05). miR-448, let-7b, miR-540, miR-296, miR-880, miR-200a, miR-500, miR-10b, miR-336, miR-30d, miR-208, let-7e, miR-142-5p, miR-874, miR-375, miR-879, miR-501, and miR-188 were upregulated, while miR-301b, miR-134, and miR-652 were downregulated in TMH group (Table 5).

### 3.6. miRNA Q-PCR Validation

**Figure 2:**
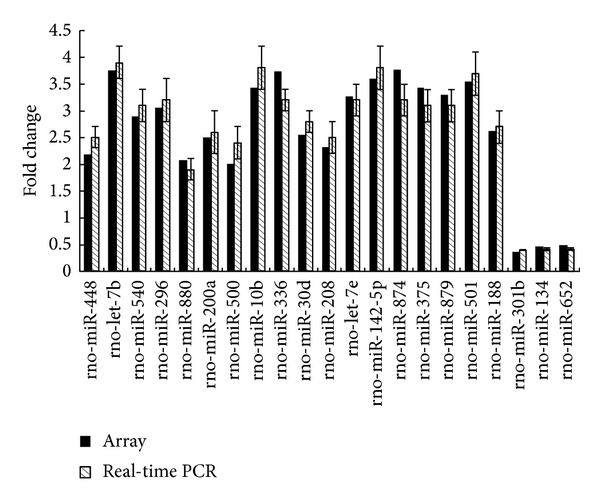
Differential miRNAs expression in gene array and Q-PCR.

To validate the microarray results, all of the miRNAs with differential expression were selected for Q-PCR quantification. All miRNA expression levels obtained by Q-PCR were similar to those observed by microarray analysis (Figure 2).

## 4. Discussion

In this study, we found that the treatment of TianMai Xiaoke Tablet to DM rats significantly reduced fasting blood glucose, fasting insulin, and HOMA-IR. Our results suggest that TianMai Xiaoke Tablet can moderate glucose and ameliorate oral glucose tolerance and insulin resistance. The main integrant of TianMai Xiaoke Tablet is chromium picolinate. During the experiment, no rats in the TML and TMH groups died. So, it is safe for rats to take TianMai Xiaoke Tablet. Rhodes et al. gave rodents chromium picolinate as 30000-fold of the adults dose for 13 weeks. No effect was observed on body weight gain or survival of rodents. No compound-related changes in hematology and clinical chemistry parameters were observed. There were no histopathological lesions attributed to chromium picolinate in rats or mice [16]. Trivalent chromium is an essential trace element involved in carbohydrate metabolism. Chromium deficiency has been considered as a possible risk factor for the development of diabetes [17]. Administration of chromium to patients with diabetes has beneficial effects in glycemic control [18]. However, a considerable number of studies have evaluated chromium in clinical research trials over the past 40 years. Some researches reported that chromium picolinate does not improve blood glucose in diabetic patients [19, 20]. But more recent trials evaluating chromium supplementation found that chromium picolinate/biotin combination, administered as an adjuvant to current prescription antidiabetic medication, can improve HbA1c and fasting blood glucose in overweight-to-obese individuals with type 2 diabetes, especially in those with poor glycemic control on oral therapy [21]. Martin et al. found that chromium picolinate supplementation had significant improvement in insulin sensitivity [22]. TM can reduce new-diagnostic type 2 diabetic patients HbA1c and increase serum chromium up to 30% [5]. Such different conclusion may be attributed to the lack of more precise glucose metabolism assessment, usage of different dose, and formulations and heterogeneous study populations.

In gene array and real-time PCR experiment, we found that TM could increase the expression of miR-375 in islet of diabetic rats. miR-375 is a regulator in the process of exocytosis of insulin during glucose-stimulated insulin release. It is highly expressed in pancreatic islets. Poy et al. found that inhibition of endogenous miR-375 function enhanced insulin secretion [23]. In addition, mice lacking miR375 (375KO) are hyperglycemic and exhibit increased total pancreatic alpha-cell numbers and decreased pancreatic beta-cell mass [24]. PDX-1 (3′-phosphoinositide-dependent protein kinase-1) is one of the validated target genes of miR-375. Ouaamari et al. found that miR-375 acts as a direct function with the 3′ untranslated region (3′UTR) of PDK1 mRNA, thus decreasing PDK1 protein, and it may impact on cell proliferation given its key role in the PI 3-kinase/PKB cascade. The expression of miR-375 is decreased in diabetic Goto-Kakizaki (GK) rats, compared with Wistar rats [25].

Moreover, TM could increase the expression of miR-30d in islet of diabetic rats. Tang et al. found that overexpression of miR-30d increased insulin gene expression, while inhibition of miR-30d abolished glucose stimulation of insulin expression. These data suggest that miR-30d is important for downregulation of an unidentified transcriptional repressor(s) of the insulin gene [26].

To sum up, TM can moderate glucose metabolism and insulin sensitivity. These actions may be through activating miR-375 and miR-30d to increase insulin secretion and action.

## Figures and Tables

**Table 1:** Composition of normal and high-fat diet.

Integrant	Normal diet (%)	High-fat diet (%)
Corn	73.5	57.9
Wheat bran	20	15.8
Fishmeal	5	3.9
Flour	1	0.8
Salt	0.5	0.4
Cholesterol	0	1
Bile salt	0	0.2
Yolk powder	0	10
Lard	0	10

**Table 2:** Body weight (g) of rats during 8 weeks.

Groups	Week 0	Week 2	Week 4	Week 6	Week 8
Control	352.0 ± 4.7	361.1 ± 5.7	370.6 ± 6.7	379.4 ± 5.8	391.8 ± 6.2
DM	347.2 ± 5.1	350.3 ± 6.2*	353.8 ± 5.8**	353.9 ± 6.9**	356.4 ± 8.3**
TML	345.0 ± 2.9	347.8 ± 3.5*	350.4 ± 5.9**	350.8 ± 4.7**	355.3 ± 7.4**
TMH	352.1 ± 6.3	355.2 ± 7.2*	358.3 ± 6.4**	360.8 ± 5.8**	358.7 ± 6.3**

TML: low dose of TianMai Xiaoke Tablet; TMH: high dose of TianMai Xiaoke Tablet.

Data represent mean ± S.D. (*n* = 8).

*P < 0.05, **P < 0.01 versus the control group.

**Table 3:** Fasting blood glucose (mmol/L) of rats during 8 weeks.

Groups	Week 0	Week 2	Week 4	Week 6	Week 8
Control	6.1 ± 0.7	6.3 ± 0.4	6.4 ± 0.5	6.3 ± 0.8	6.2 ± 0.7
DM	23.7 ± 3.2**	24.1 ± 2.5**	25.3 ± 3.1**	24.8 ± 4.8**	23.6 ± 4.3**
TML	24.4 ± 4.1**	18.0 ± 3.5^∗∗#^	19.6 ± 2.6^∗∗#^	18.5 ± 3.4^∗∗#^	19.4 ± 4.1^∗∗#^
TMH	22.5 ± 4.2**	15.4 ± 5.3^∗∗#^	14.5 ± 3.5^∗∗#^	16.3 ± 4.3^∗∗#^	15.3 ± 5.3^∗∗#^

TML: low dose of TianMai Xiaoke Tablet; TMH: high dose of TianMai Xiaoke Tablet.

Data represent mean ± S.D. (*n* = 8).

**P < 0.01 versus the control group. ^#^ *P* < 0.05 versus DM group.

**Table 4:** Fasting insulin (ng/mL) and HOMA-IR levels.

Groups	FINS (*μ*IU/mL)	HOMA-IR
Control	10.74 ± 2.50	5.96 ± 0.89
DM	31.90 ± 4.68**	33.46 ± 8.30**
TML	18.74 ± 5.38^∗##^	16.12 ± 4.73^∗∗##^
TMH	15.79 ± 3.75^∗##^	15.83 ± 4.87^∗∗##^

TML: low dose of TianMai Xiaoke Tablet; TMH: high dose of TianMai Xiaoke Tablet.

Data represent mean ± S.D. (*n* = 8).

*P < 0.05, **P < 0.01 versus the control group; ^##^ *P* < 0.01 versus DM group.

**Table 5:** Differentially expressed miRNAs (fold > 2, *P* < 0.05).

rno miRNA gene	Fold change	*P* value	Chromosomal location	Mature sequence
rno-miR-448	2.175	0.04844	Xq14	UUGCAUAUGUAGGAUGUCCCA
rno-let-7b	3.740	0.02207	7q34	UGAGGUAGUAGGUUGUGUGGU
rno-miR-540	2.899	0.04278	6q32	AGGUCAGAGGUCGAUCCUG
rno-miR-296	3.054	0.02955	3q42	GAGGGUUGGGUGGAGGCUCUCC
rno-miR-880	2.069	0.03130	Xq37	UACUCCAUUCAUUCUGAGUAG
rno-miR-200a	2.493	0.01933	5q36	UAACACUGUCUGGUAACGAUG
rno-miR-500	2.010	0.01848	Xq13	AAUGCACCUGGGCAAGGGUUCA
rno-miR-10b	3.413	0.04138	3q23	CCCUGUAGAACCGAAUUUGUG
rno-miR-336	3.721	0.00746	10q22	UCACCCUUCCAUAUCUAGUC
rno-miR-30d	2.531	0.01836	7q34	UGUAAACAUCCCCGACUGGAA
rno-miR-208	2.300	0.46728	15p13	AUAAGACGAGCAAAAAGCUUG
rno-let-7e	3.268	0.02307	1q12	UGAGGUAGGAGGUUGUAUAGU
rno-miR-142-5p	3.582	0.00483	10q26	CAUAAAGUAGAAAGCACUACU
rno-miR-874	3.751	0.01429	17p14	CUGCCCUGGCCCGAGGGACCG
rno-miR-375	3.412	0.02933	9q33	UUUGUUCGUUCGGCUCGCGUG
rno-miR-879	3.299	0.00634	4q12	AGAGGCUUAUAGCUCUAAGC
rno-miR-501	3.534	0.01714	Xq13	AAUCCUUUGUCCCUGGGUGAA
rno-miR-188	2.609	0.03009	Xq13	CAUCCCUUGCAUGGUGGAGGG
rno-miR-301b	0.354	0.05323	11q23	CAGUGCAAUGGUAUUGUCAAAG
rno-miR-134	0.458	0.00298	6q32	UGUGACUGGUUGACCAGAGGGG
rno-miR-652	0.477	0.01357	Xq14	AAUGGCGCCACUAGGGUUGU
